# Esophageal Adenocarcinoma and Its Rare Association with Barrett’s Esophagus in Henan, China

**DOI:** 10.1371/journal.pone.0110348

**Published:** 2014-10-15

**Authors:** Shuzheng Liu, James Y. Dai, Lena Yao, Xiaohong Li, Brian Reid, Steve Self, Jie Ma, Yuxi Chang, Shixian Feng, Jean de Dieu Tapsoba, Xin Sun, Xibin Sun

**Affiliations:** 1 Henan Office for Cancer Research and Control, Henan Cancer Hospital, Zhengzhou, Henan Province, China; 2 Vaccine and Infectious Diseases and Public Health Science Division, Fred Hutchinson Cancer Research Center, Seattle, Washington, United States of America; 3 Divisions of Human Biology and Public Health Sciences, Fred Hutchinson Cancer Research Center, Seattle, Washington, United States of America; 4 Department of Molecular pathology, Henan Cancer Hospital, Zhengzhou, Henan Province, China; 5 Henan Center for Disease Control and Prevention, Zhengzhou, Henan Province, China; 6 Institute of occupational Health and Poison Control, Chinese Center for Disease Control and Prevention, Beijing, China; Vanderbilt University Medical Center, United States of America

## Abstract

Incidence of esophageal adenocarcinoma (EAC) has increased sharply in Western Europe and United States over the past three decades. Nearly all cases of EAC in the west are thought to be associated with Barrett’s esophagus (BE) at the time of diagnosis. Regions in the Henan province of China have one of world’s highest incidences of esophageal cancer, yet recent temporal trends in the relative rates of EAC with respect to esophageal squamous-cell carcinoma (ESCC), as well as its association with Barrett’s esophagus (BE), have not been reported. In this report, we present large-scale longitudinal clinical and histological data on 5401 esophageal cancers (EC) patients diagnosed during the recent 10-year period (2002–2011) at Henan Cancer Hospital, China. All 217 esophageal adenocarcinoma (EAC) patients from these 5401 EC patients were examined to better understand the relationship between Barrett’s esophagus (BE) and EAC. We found that EAC was relatively rare and accounted for approximately 5% of all esophageal cancers each year during 2002–2011. There is no evidence of significant temporal trends in the rate of EAC relative to ESCC. Only 10 out of 217 (4.6%) EAC cases were detected to have any evidence of Barrett’s esophagus. This result raises the possibility of a different etiological basis for EAC in China motivating more detailed epidemiological, clinical and molecular characterization of EAC in China in order to better understand the neoplastic development of EAC.

## Introduction

Esophageal carcinoma (EC) is the eighth most common cancer and the sixth leading cause of cancer-related mortality worldwide [1]. The incidence of EC varies widely by geographic region [2]. In the United States (US) and Western Europe, the predominant histological type of EC has shifted over the past four decades from esophageal squamous-cell carcinoma (ESCC) to esophageal adenocarcinoma (EAC), as the incidence of EAC has increased 5–6 fold while the incidence of ESCC has remained relatively stable [3], [4], [5], [6]. This increase in EAC began in the early 1970s in both male and female, but seems more pronounced in white males. In the US, the rate of increasing in the age-adjusted incidence of EAC reached nearly 10% per year among men [3]. This rapid and substantial shift in the incidence of EAC relative to ESCC in western countries suggests the possibility of changing environmental factors and microorganisms as etiological agents, which have yet to be determined.

A majority of reported cases of EAC are believed to arise from Barrett’s esophagus (BE), a condition of esophagus characterized by a columnar-cell metaplasia that replaces the native squamous-cell epithelium [7], [8], [9], [10]. BE is considered to be a complication of gastroesophageal reflux disease (GERD), which is also significantly associated with the risk for EAC [11]. The paradigm is that BE arises from an adaptation to the harsh intra-esophageal environment of chronic GERD, and predisposes to EAC. Other established risk factors for EAC include obesity, smoking, diet with low fruit and vegetable consumption, suggesting that changes in behavior and life style in western countries, prior to 1970’s and possibly still ongoing, may explain part of the rising incidence of EAC [12], [13].

Epidemiology of EAC and BE in eastern Asian countries is quite different. In Japan, the prevalence of BE is much lower than that in the West, and EAC is still rare, though a slight increase in both EAC and ESCC has been reported [14], [15]. In China and its surrounding areas, ESCC is still the predominant type and the pattern of sharply increasing incidence of EAC has not been observed [16], [17], [18], [19]. The ratio of EAC and ESCC did not increase in the past two decades in a study from Taiwan reported in 2010 [18] and the incidence of EAC in Hong Kong was reported to decrease from 1984–2003 [16]. In northern China, areas such as Lin County, Henan Province have one of world’s highest rates of ESCC [20]. In Linzhou city, the incidence of ESCC has been reported to be on the rise but EAC incidence remains low from 2003–2009 [19]. There is a recent hospital report of a largely stable trend in the ratio of EAC relative to ESCC in Cixian, Hebei Province from 1982 to 2005 [17].

The data on Barrett’s esophagus and its association with esophageal adenocarcinoma in China prior to 2007 are difficult to interpret due to lack of standard criteria for endoscopic classification of the gastro-esophageal junction and BE diagnosis in China [21]. Adopting the American Gastroenterological Association Guidelines, several studies recently showed striking differences in the occurrence and nature of Barrett’s esophagus in China compared to those of western countries [21]. Specifically, although columnar-lined esophagus is common, but incidence of BE with intestinal metaplasia is still rare; most cases of BE were “short segment” while the “long segment” BE was uncommon. Preliminary data from China also reported that age, hiatal hernia, tobacco and alcohol abuse, but not obesity, may be the major risk factors for BE in China [21], suggesting that pathogenesis mechanisms may be different from those seen in the West.

In western countries, BE is generally considered to be “the” premalignant condition for EAC. The prevalence of BE detection in EAC patients under surgical resection was reported to vary from 37% to 97% [9], [22], [23], [24]. The failure to identify BE in some EAC patients has been attributed to large tumors that mask BE. This interpretation is supported by reports that chemotherapy could unmask BE [24], and a higher prevalence of BE observed in smaller tumors diagnosed at earlier stages [23]. Since EAC is rare in China, systematically examining BE in surgical resection specimens of EAC patients is an effective approach to study relationship between BE and EAC in the Chinese population.

China has been going through remarkable economic growth since end of the 70s. The social and economic conditions for Chinese population have been markedly improved, triggering changes in lifestyle behaviors such as diet, alcohol and tobacco consumption. It is not clear from existing data whether the epidemic switch from ESCC to EAC that occurred in the West, possibly strongly affected by lifestyle choices and mediated by GERD and BE, or host microorganisms such as H. pylori and HPV, is on the Chinese horizon.

In this report we present large-scale longitudinal clinical and histological data on 5401 EC patients diagnosed during the recent 10-year period (2002–2011) at Henan Cancer Hospital, China, where it is known to be a high risk region for EC. These data were developed by a comprehensive and standardized review of tissue from all cases in the 10-year series applying American Gastroenterological Association Guidelines for tumor classification. This series of newly diagnosed cases of esophageal cancer represents the largest in China to date that is uniformly and rigorously assessed by a group of oncologists and pathologists, systematically reviewing pathological reports, repeating the process of pathology diagnosis for some cases to classify tumor site and stage based on International Classification of Diseases for Oncology (Third Edition) and American Joint Committee on Cancer (Sixth Edition). Admittedly, these data are derived from records of a single hospital, therefore cannot be used to compute absolute cancer incidence as data from a well-collated cancer registry. However there are no cancer registry data in Henan Province as a whole, existing cancer registry data are at best scattered, poor in completeness and accuracy, and lacking consistency in tumor classification and diagnosis. Therefore, the hospital-based data we reported represent the best quality possible for this region of China. Moreover, the main purpose of this paper is to discover the trend of the ratio between EAC and ESCC for the region, which should be consistent in hospital reports and the population registry, if there is one, since patient referral should be affected by the histology subtype diagnosed later.

Furthermore, to better understand the relationship between BE and EAC in this new study population, evidence of BE was also examined from the tumor resection specimen among 217 EAC patients. Evaluating the differences of BE/EAC between western countries and China at the population level provides a basis for designing future studies to better understand the overall mechanisms of development of EAC that leads to effective early detection, prevention and therapy.

## Patients and Methods

Henan Cancer Hospital, located in Zhengzhou City, Henan Province, China, is one of the largest oncology hospitals in the region. The hospital has 3000 beds and received more than 77,000 in-patients with cancer in 2013. About 99% of patients were from Henan Province with approximately one hundred million residents. The Institutional Review Board of Henan Cancer Hospital approved the study. Because this is a retrospective analysis of our clinical and pathological database, the consent of study participants could not be obtained and therefore the data were analyzed anonymously. Data on all esophageal cancer patients enrolled from January 1, 2002 to December 31, 2011 were extracted from database of pathological diagnosis in Henan Cancer Hospital and the subset of all newly diagnosed patients was identified for inclusion in the study. Pathology tissue specimens were obtained on all subjects and were verified to have been collected prior to delivery of any chemotherapy or radiotherapy. Information obtained from the clinical and pathological database included patient name, year, age at diagnosis, gender, tumor site and tumor type and stage as determined at the time of pathological diagnosis. The identification of tumor sites and categories was based on International Classification of Diseases for Oncology (Third Edition). The procedure of sample collection form the clinical database is as following: (1) obtain pathology reports that phenotype contains “tumor” or “cancer”; and location contains “esophagus”, “proximal stomach”, or “cardia”; (2) exclude cases that have received radiation or chemo therapy or other intervention therapy; (3) select the cases that reported the tumor locations in esophagus and cardia, esophagus and stomach or cardia and recheck the original histology inspection sheet and sections of the case by the pathologist for further verification of the location of tumor; (4) drop the non-esophagus cancer cases to get the final dataset for the study. All esophagus cancers corresponded to the ICD code C15 and were classified into three categories: squamous cell carcinoma (morphology code 8050-8083), adenocarcinoma (morphology code 8140-8576) and other types of malignant tumor (morphology codes do not belong to 8050-8083 or 8140-8576). Tumor staging from 0–IV was based on tumor-node-metastasis (TNM) classification by American Joint Committee on Cancer (Sixth Edition).

The pathological tissue slides of the patients who were diagnosed with esophageal adenocarcinoma were re-evaluated by the pathologist for existence of Barrett’s esophagus. The location of Barrett’s tissue in the esophagus, if confirmed, was based on information of the location of biopsies (low, middle, upper part of esophagus). The detection of Barrett’s esophagus is based on practice guidelines of American College of Gastroenterology [25] histological existence of intestinal metaplasia and goblet cells.

Descriptive summary statistics and significance testing for two-sample comparison and trend in years were performed using the statistical software R. Differences were considered to be statistically significant at a 2-sided p-value<0.05.

## Results

We first report the distribution pattern of EAC and ESCC stratified by age and gender in the study. Table 1 summarizes the distribution of the number of new esophageal cancer patients diagnosed as ESCC, EAC and other EC by gender and age categories from 2002 to 2011. Approximately 2/3 of ESCC and other EC patients are male, and EAC patients are somewhat more likely to be male compared to ESCC patients (77% versus 68%, p = 0.009). The male/female ratio in the EAC subjects of 3∶1 is much lower than what has been reported in the US population (up to 8∶1 in whites) [3], [5], but consistent with what were reported in other Chinese populations [16], [18], and similar to that in the UK populations (4∶1) [26], [27]. It was suggested that the gender difference may not be all explained by differential risk factors in males and females [28]. There is a small but statistically significant (p<0.001) difference in the median age at diagnosis between ESCC (median 60, IQR = 12) and EAC (median = 62, IQR = 13) and this difference was consistent for both male and female cases.

**Table 1:** Distribution of esophageal squamous cell carcinoma (ESCC) and esophageal adenocarcinoma (EAC) by sex and age.

	ESCC *n(%)*	EAC *n(%)*	Other *n(%)*	Total *n(%)*	P-value
Total patients	5013 (100)	217 (100)	171 (100)	5401 (100)	
Male	3403 (67.9)	166 (76.5)	112 (65.5)	3681 (68.2)	
Female	1610 (32.1)	51 (23.5)	59 (34.50)	1720 (31.8)	0.009‡
Age					
Median (IQR)	60 (12)	62 (13)	60 (9)	60 (11)	<0.001±
**By sex and age**					
Male					
Age					
Median (IQR)	60 (11.5)	62 (12.8)	59 (8)	60 (12)	<0.001±
≤30	3 (0.1)	0 (0)	0 (0)	3 (0.1)	
30–40	55 (1.6)	0 (0)	1 (0.9)	56 (1.5)	
40–50	471 (13.8)	22 (13.3)	7 (6.3)	500 (13.6)	
50–60	1358 (39.9)	54 (32.5)	60 (53.6)	1472 (40.0)	
60–70	1153 (33.9)	62 (37.4)	34 (30.4)	1249 (33.9)	
>70	363 (10.7)	28 (16.9)	10 (8.9)	401 (10.9)	
Female					
Age					
Median (IQR)	61 (11)	62 (11)	62 (7.50)	61 (11)	0.042±
≤30	0 (0.0)	0 (0)	0 (0)	0 (0.0)	
30–40	19 (1.2)	0 (0)	1 (1.7)	20 (1.2)	
40–50	137 (8.5)	2 (3.9)	6 (10.2)	145 (8.4)	
50–60	602 (37.4)	17 (33.3)	18 (30.5)	637 (37.0)	
60–70	638 (39.6)	23 (45.1)	29 (49.2)	690 (40.1)	
>70	214 (13.3)	9 (17.7)	5 (8.5)	228 (13.3)	

IQR: Interquartile range.

‡ P-value associated with the test for equal proportions of men between EAC and ESCC patients.

± P-value associated with the comparison of median ages between EAC and ESCC patients.

**Figure 1:**
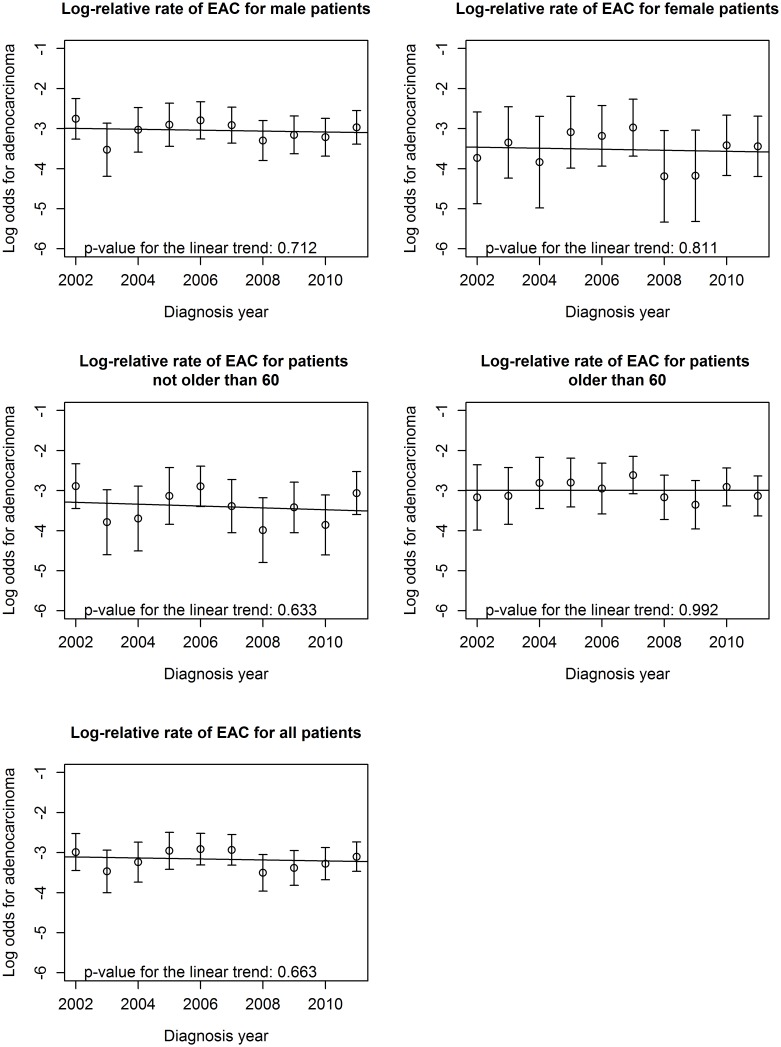
Log odds for esophageal adenocarcinoma among esophageal cancer over the study period (2002–2011).

We evaluate temporal changes of incidence of EAC and ESCC in the study region. Table 2 shows the number of new patients yearly diagnosed in each type of ESCC, EAC and other EC, as well as the percentage of EAC diagnosed out of total EC patients each year. The absolute number of ESCC and EAC patients increases gradually during the 10-year period but the percentage of EAC remains stable over time at 5%. Figure 1 shows the estimated rate of EAC relative to that for non-EAC cases plotted on the log scale together with 95% confidence intervals around each log-relative rate. The rates were calculated for overall patients, male or female patients as well as for patients younger or older than the overall median age (60). Formal testing of a linear trend across years in these log relative rates was non-significant (p = 0.66) in overall patients. Similarly, there was significant linear trend in these rates for patients who were male (p = 0.71), female (p = 0.81), not older than 60 (p = 0.63) or older than 60 (p = 0.99). Tests for trends over time were also performed for age at diagnosis and gender distribution. The mean of age at diagnosis for EC patients increases from 57.9 in 2002 to 61.6 in 2011, with a significant linear trend (p = 0.0007). This trend is consistent in ESCC patients (p = 0.0005), but not significant in EAC patients (p = 0.255). There was no temporal change in the gender composition of either of the two cancer types (p = 0.972 for ESCC and p = 0.663 for EAC).

**Table 2:** Distribution of esophageal squamous cell carcinoma (ESCC) and esophageal adenocarcinoma (EAC) by year.

Year	ESCC	EAC *n*	Other *n*	Total *n*	% EAC	P-value
2002	360	19	17	396	4.80	
2003	439	14	10	463	3.02	
2004	392	16	16	424	3.77	
2005	349	19	16	384	4.95	
2006	460	26	20	506	5.14	
2007	507	28	18	553	5.06	
2008	618	19	14	651	2.92	
2009	597	21	22	640	3.28	
2010	639	25	23	687	3.64	
2011	652	30	15	697	4.30	0.663§

§ P-value associated with the test for a linear trend across years in log relative rate of EAC.

Table 3 shows the distribution of tumor stage at diagnosis for ESCC, EAC and other EC. The newly diagnosed ESCC and EAC patients were predominantly in stage II and III. Relatively speaking, EAC patients had a higher percentage of stage III or higher than ESCC patients (37% vs 25%, p = 0.0001). Patients with other EC types had more early-stage (stage 0 and 1) tumors compared to ESCC and EAC. Tests for trends over time in the proportion of patients with stage III or higher at diagnosis were not significant for either EAC (p = 0.236) or ESCC (p = 0.168). The proportion of patients with stage III or higher among male ESCC patients is higher than it is among female ESCC patients (27% vs 21%, p<0.0001). Similarly, the proportion of patients with stage III or higher among male EAC patients is higher than it among female EAC patients, albeit not significant (40% vs 29%, p = 0.24).

**Table 3:** Distribution of esophageal squamous cell carcinoma (ESCC) and esophageal adenocarcinoma (EAC) by tumor stage and sex.

	ESCC *n(%)*	EAC *n(%)*	Other *n(%)*	Total *n(%)*	EAC with BE *n*
**Tumor stage**					
0	53 (1.06)	0 (0)	27 (15.79)	80 (1.48)	0
I	454 (9.06)	14 (6.45)	31 (18.13)	499 (9.24)	1
II	3230 (64.43)	122 (56.22)	82 (47.95)	3434 (63.58)	6
III	1276 (25.45)	81 (37.33)	30 (17.54)	1387 (25.68)	3
IV	0 (0)	0 (0)	1 (0.58)	1 (0.02)	0
Total	5013	217	171	5401	10
**Sex**					
Male					8
0	26 (0.76)	0 (0)	14 (12.50)	40 (1.09)	0
I	288 (8.46)	12 (7.23)	21 (18.75)	321 (8.72)	1
II	2157 (63.39)	88 (53.01)	53 (47.32)	2298 (62.43)	5
III	932 (27.39)	66 (39.76)	23 (20.54)	1021 (27.74)	2
IV	0 (0)	0 (0)	1 (0.89)	1 (0.03)	0
Female					2
0	27 (1.68)	0 (0)	13 (22.03)	40 (2.33)	0
I	166 (10.31)	2 (3.92)	10 (16.95)	178 (10.35)	0
II	1073 (66.65)	34 (66.67)	29 (49.15)	1136 (66.05)	1
III	344 (21.37)	15 (29.41)	7 (11.86)	366 (21.28)	1
IV	0 (0)	0 (0)	0 (0)	0	0

Numbers in parentheses () are the column percentages.

The majority of 217 EAC located in the middle third of the esophagus part (65%) and some located in the lower third (32%). There is no significant difference of the distribution of tumor locations between men and women (p = 0.61). A total of 1305 biopsy specimen from 217 EAC patients were examined. On average 6 biopsies per patient were examined for the presence of BE, based on histological and pathological slides, pathology report, endoscopic image and clinical. Among the 217 subjects with diagnoses of EAC, only a total 10 patients (4.6%) had BE detection in tumor biopsies, all of which located in the lower third of the esophagus. This rate is substantially lower than what were observed in EAC biopsies in western countries previously reported (37%–97%) [9], [22], [23], [24]. It is interesting to note that 7 out of 10 EAC patients with BE were in the early tumor stages and 8 of them were from male patients.

## Discussion

We presented results based on data from 5,401 newly diagnosed esophageal cancer cases in Henan Cancer Hospital, China from 2002–2011. To our knowledge this is the most recent comprehensive report on the trend of EAC and ESCC in Henan Province, a region of the highest risk in esophageal cancer in China. The strength of our data stems from the rigorous and uniform retrospective assessment of esophageal cancer cases accrued over a ten-year period using international classification standards for tumor site and stage. A unique contribution of this work is the detailed examination of the association between Barrett’s esophagus and esophageal adenocarcinoma in China.

We observed that the absolute number of new cases of EC diagnoses at Henan Cancer Hospital is substantially increasing over time yet this trend is not likely due to large increases in the population rates of EC. The likely causes of this increase are the increasing use of upper gastrointestinal endoscopy, changes in referral patterns for EC, and simply expanded capacity of the hospital for examining more patients. Although the absolute rates observed for EAC and ESCC diagnoses do not reflect population incidence, we believe that the observed relative rates reliably reflect population relative rates as the suite of signs/symptoms that initiate a diagnostic evaluation are essentially the same for EAC and for ESCC.

Our data provide unequivocal evidence of the rare occurrence of EAC and the stable ratio of EAC to ESCC throughout the 10 year period, consistent with previous reports in China [17], [19]. This is strikingly different from the epidemic switch observed in western countries. Notably, the significant rising trend of EAC in the US was observed since 1970’s with this increase occurring predominantly in white males. In an earlier report using population-based US cancer registry data, the incidence of EAC from 1976 to 1987 in white men doubled while that of ESSC remained stable [3]. Similar cancer registry data from 1975 to 2000 showed the incidence of EAC steadily rising from 3.8 to 23.3 per million populations [4]. In a separate study of New South Wales cancer registry, the EA incidence rate in male was relative steady from 1972 to 1981 then rose sharply after 1981, in a pattern resembling that observed in US [6]. It was speculated that risk factors driving this increase may include obesity, tobacco and alcohol consumption and possible falling rate of Helicobacter pylori infection [6]. Although rates of EAC in China are currently stable, it is not clear whether future rates will undergo dramatic increases as has been observed in the west.

The social and economic conditions have rapidly changed in China over the past 30 years, including some that align with major western risk factors for esophageal cancer. Data from a national census report [29] showed that, fat intake per day increased 30–40% from 1982 to 2002; cigarette consumption increased 17.4% from 2002 to 2006; proportion of overweight adults has increased 39% and obese adult increased 97% from 1992 to 2002, largely due to dietary change and decreased physical exercise [29]. Some of these risk factors, e.g. smoking, are thought to contribute the increased incidence of ESCC observed in Taiwan [18]. Obesity has a consistent historical association with EAC in the west [30]. The direction of these behavioral and lifestyle changes does not explain the rare and steady occurrence of EAC in our data, though it is not clear whether there is a time lag of life-style change exerting effect on EAC epidemiology. It is also possible that these changes of life-style factors have not yet reached the level to trigger the epidemiologic switch of the esophageal neoplastic process. On the other hand, the infection rates of H-pylori in China remain high, 40%–60% depending on areas of serum sampling [31], which may also contribute to difference of EAC epidemiology between China and western countries. Prevalence of GERD, the strongest risk factor of EAC, was reported to be 3.1% in a Chinese population-based survey recently [32], much lower than the reported 10–20% in the west [33], [34], [35]. In other parts of Asia, there is a rising trend of GERD since late 90s. As discussed before [36], adoption of Western lifestyles in Asia was predicted to trigger the rise of EAC incidence. However the data from our study, as well as other recent reports in Asia, suggests that such rise has not yet occurred.

The other important discovery from our data is the rare occurrence of BE among Chinese cases of EAC. It may be that this finding is due in part to systematic under ascertainment of BE. The practice guidelines for the diagnosis of BE have been evolving and there is currently no universal agreement on diagnostic criteria [25]. The American Gastroenterological Association and the American College of gastroenterology currently recommends that although columnar-type mucosa can be recognized during endoscopy, the presence of intestinal metaplasia must be confirmed by biopsy before rendering a diagnosis of BE [25]. We have adopted this definition of BE and made rigorous efforts to examine histological evidence of intestinal metaplasia in multiple biopsies among all EAC patients. However, even if there are some subtle biases in the application of diagnostic criteria for BE in this study, they are unlikely to fully explain the dramatic difference in estimated prevalence of BE among EAC cases and that among EAC cases from the west.

The relationship between BE and EAC has been intensively studied in western countries and there is a wealth of clinical and epidemiological evidence supporting the hypothesis that BE is a precursor lesion of EAC [12],[37]. However many EAC cases do not have previous clinical diagnoses of BE and, when examining adjacent tissue samples around EAC tumor samples, not all EAC cases have evidence of BE as characterized by intestinal metaplasia [9], [22], [23], [24]. The lack of prior clinical diagnosis of BE among EAC cases may simply be due to the lack of an effective means for detection of BE prior to the occurrence of the symptoms of EA [37]. Moreover, the lack of evidence for BE in adjacent tissue samples around EAC may be due to overgrowth of the tumor obscuring BE, incomplete spatial sampling of tissue or other methodological issues regarding diagnostic criteria. Thus, the current data on BE and EAC co-occurrence may somewhat underestimate the true association between these two conditions. In a rigorous Swedish case-control study, the association of GERD and EAC in patients without BE was equally strong as the association in patients with BE, supporting the hypothesis that gastro-esophageal reflux, rather than Barrett’s esophagus, may be the crucial causal factor for EAC [11]. Thus, clinical and epidemiological data from multiple sources suggest that BE may be a common precursor in the west, but perhaps not a necessary step in the evolution of EAC [11], [38].

The association between BE and EAC has not previously been carefully examined in Chinese populations, possibly due to low incidence of EAC. Despite the rigorous effort to detect BE among tumor and tumor-adjacent tissue, we found a strikingly low rate (4.6%) of detection of BE in 217 EAC samples cumulated in 2002–2011. We also found the majority of Chinese EAC were located in the middle, but not the lower third of the esophagus typically found in the west, Moreover, epidemiological data suggest that the prevalence of BE remains rare in China, ranging from 0.06% in the general population to <2% in referral patients [21], whereas in a Swedish study the BE prevalence in general population is estimated 1.6% and the reported prevalence in US cohorts ranges from 5%–25% [39], [40], [41], [42]. This all suggests that, despite the prevailing theory in the west that long-term GERD leads to BE which is an obligate precursor lesion to EAC, BE may not be a necessary step in the development of EAC in China.

Perhaps more intriguing is the notion that there are two types of EAC, a “sporadic” form that arises rarely in a population and a second form with population rates that are driven by environmental insults that are initially manifest as BE. The former type is seen predominately in China as well as in some small fraction of EAC cases in the west that are not associated with BE while the latter, driven by environmental factors, is currently seen rarely in China but quite frequently in the west. In this hypothesized model, the large historical increases in EAC incidence in the west have occurred in this second form which adds to a smaller but steady occurrence of sporadic cases.

Taking collectively, our results reveal prominent differences in the epidemiological characteristics of EAC between Henan, China and western countries. Additional epidemiological and clinical data, as well as molecular and genomic characterizations of EAC in China, in comparison to similar characterizations of EAC in the west, could provide important knowledge regarding the etiology of EAC, especially when these comparisons are stratified by EAC with and without BE.

## Supporting Information

Table S1 **The incidence data of esophageal cancer in Henan Cancer Hospital (2002–2011) including histology type, demographics and clinical stages.**
(XLS)
