# Correction: Esophageal Adenocarcinoma and Its Rare Association with Barrett's Esophagus in Henan, China

**DOI:** 10.1371/journal.pone.0127135

**Published:** 2015-04-28

**Authors:** Shuzheng Liu, James Y. Dai, Lena Yao, Xiaohong Li, Brian Reid, Steve Self, Jie Ma, Yuxi Chang, Shixian Feng, Jean de Dieu Tapsoba, Xin Sun, Xibin Sun

The tenth author’s last name is improperly indicated. The correct name is: Jean de Dieu Tapsoba. The correct abbreviation is: Tapsoba JD.
